# Lactylation: From Molecular Insights to Disease Relevance

**DOI:** 10.3390/biom15060810

**Published:** 2025-06-03

**Authors:** Yao Xu, Lu Zhang, Dong Shang, Hong Xiang

**Affiliations:** 1 Laboratory of Integrative Medicine, First Affiliated Hospital of Dalian Medical University, Dalian 116011, China; xuy05@dmu.edu.cn; 2 Department of General Surgery, First Affiliated Hospital of Dalian Medical University, Dalian 116011, China; 3 Department of Pharmacy, First Affiliated Hospital of Dalian Medical University, Dalian 116011, China; zhangl33@dmu.edu.cn; 4 Department of Pharmacy, Dalian Medical University, Dalian 116044, China

**Keywords:** lactylation, tumor, inflammation, metabolic reprogramming

## Abstract

Lactylation, referring to the covalent coupling of the lactyl group with lysine residues, is a recently defined post-translational modification. It has been demonstrated that lactylation can alter protein transcription, thereby affecting the transmission of genetic information and ultimately exerting diverse effects on health and diseases. Here, we review the existing literature and summarize the characteristics and mechanisms of lactylation on both histone and non-histone proteins. We hope to explore lactylation targets for different diseases, thus providing potential clues for new therapeutic strategies.

## 1. Introduction

Lactate has been regarded as a metabolic waste product under hypoxic conditions with multiple harmful effects. Unexpectedly, in 2019, Zhang et al. [1] demonstrated for the first time the new role of lactate in epigenetic regulation, namely histone lysine lactylation (Kla). After that, studies have identified various non-histone Kla sites and reported their impacts on a broader range of biological processes. This review summarized the newly discovered Kla sites on histone and non-histone proteins with specific regulatory mechanisms, which helps to better understand the function of Kla and provide new potential targets for diseases.

## 2. Formation and Regulation of Kla

Lactate is the product of glycolysis and an essential substance for various physiological cellular functions, playing a regulatory role in different aspects of energy metabolism and signal transduction. It is well known that under hypoxic conditions, such as during intense exercise, muscle cells break down glucose into pyruvate via the glycolytic pathway. Due to the lack of oxygen, pyruvate cannot enter the mitochondria for aerobic oxidation and is instead reduced to lactate-by-lactate dehydrogenase (LDH). The generated lactate is released into the bloodstream and transported to the liver via circulation. In the liver, hepatocytes take up lactate, which is then converted back to pyruvate by lactate dehydrogenase. Pyruvate subsequently undergoes gluconeogenesis to synthesize glucose. This process is known as the Cori cycle [2]. In the 1920s, Otto Warburg first observed that tumors consume more glucose than surrounding normal tissues and proposed the phenomenon of aerobic glycolysis, where glucose is fermented to produce lactate rather than carbon dioxide even in the presence of oxygen. This phenomenon is now referred to as the Warburg effect [3]. Lactate transmembrane transport can be guided by monocarboxylate transporters (MCTs) to facilitate the influx and efflux of lactate across the plasma membrane, transporting lactate to tissues and organs with elevated oxidative reactions [4]. In particular, MCT1 and MCT4 have been identified. Current research has demonstrated that increased activity of MCT1-mediated lactate transport can elevate histone Kla levels [5]. MCT4 can also promote lactate efflux, thereby promoting histone Kla [6]. Lactate levels increase through the above-mentioned multiple pathways and serve as the initiating substance for protein Kla.

Lactate must first be converted into lactyl-CoA, which serves as the lactate donor for protein Kla. The enzyme catalyzing this conversion remained unidentified for some time, but recent evidence confirms that Acetyl-CoA synthetase 2 (ACSS2), as a lactyl-CoA synthetase, plays a vital role in converting lactate to lactyl-CoA [7].

“Writers” are enzymes that catalyze specific post-translational modifications (PTMs) on proteins. These enzymes recognize the target protein’s specific sequence or structural domain through their active sites and add modifying groups to the corresponding amino acid residues. After lactyl-CoA is generated, “writers” transfer the lactyl group to lysine residues on histones or non-histone proteins, altering their structure and function. Multiple “writers” have been identified, each playing unique roles in Kla. Histone acetyltransferase (p300), a multifunctional transcriptional coactivator, acts as a “writer” for Kla in the context of Yin Yang-1 (YY1) regulation [8,9]. Nijmegen Breakage Syndrome protein 1 (NBS1) is known for its role in DNA damage repair and signal transduction repair, and Tat-interactive protein 60 kDa (TIP60) functions as an NBS1 K388la “Writer” extending its biological versatility [10]. In addition, Lysine acetyltransferase 8 (KAT8) has been identified as a “writer” for pan-Kla [11].

After the lactyl group is connected to the protein, “readers” are required to specifically recognize and bind to lactylated proteins or structural domains. They interact with the modified amino acid residues and their surrounding structural environment to interpret the modification and mediate downstream biological functions. It has been demonstrated that Kla can promote the expression of the m6A reader protein YTHDF2 in tumors, thereby affecting downstream pathways to initiate biological events [12]. Additionally, Brahma-related gene 1 (Brg1) interacts with H3K18la, enriched at the promoters of metabolism-related genes, acting as a Kla “reader” to promote reprogramming [13].

**Figure 1:**
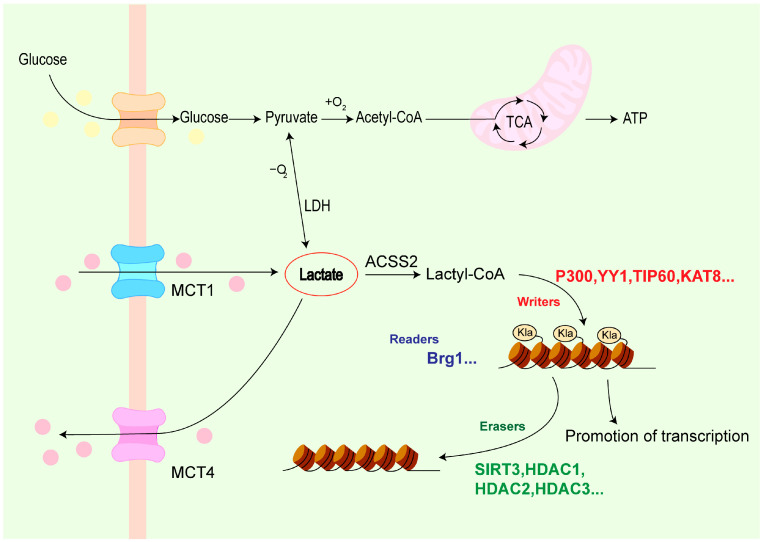
Formation and regulation of Kla. Abbreviations: ATP (Adenosine triphosphate); LDH (Lactate dehydrogenase); MCT (Monocarboxylate transporter); ACSS2 (Acetyl-CoA synthetase 2); YY1 (Yin Yang-1); TIP60 (Tat-interactive protein 60 kDa); KAT8 (Lysine acetyltransferase 8); Brg1 (Brahma-related gene 1); Kla (Lysine lactylation); SIRT3 (Sirtuin 3); HDAC (Histone deacetylase).

Finally, after the modification information is decoded and used to mediate the completion of downstream biological functions, the “eraser” (de-modification enzyme) can catalyze chemical reactions to remove the previously added Kla modification groups. Thus, it changes the properties and functional states of proteins and maintains a dynamic balance in the Kla process. Sirtuin 3 (SIRT3) acts as an “eraser” for H4K16la, counteracting Kla’s biological effects and maintaining cellular homeostasis [14]. Histone deacetylase 1 (HDAC1), HDAC2, and HDAC3 also exhibit delactylase activity [15,16]. These deacetylases play broad roles in gene expression regulation and chromatin structure maintenance. As delactylases, they fine-tune Kla modifications, ensuring precise cellular responses to internal and external stimuli while preventing dysfunction or disease due to aberrant Kla accumulation (Figure 1).

## 3. Biological Effects of Kla

**Figure 2:**
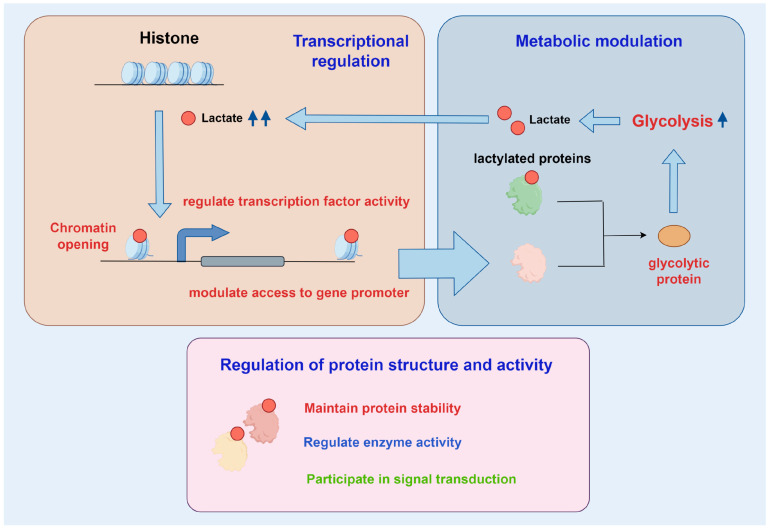
The biological effects of Kla.

With the deepening of research, the molecular mechanisms of Kla are being progressively elucidated [17]. This section summarized the biological functions of Kla from four perspectives: transcriptional regulation, metabolic modulation, protein structure and activity, and interactions between Kla and other PTMs (Figure 2). The diversity of Kla modification sites on proteins enables cells to engage in multifaceted life activities through an extensive and sophisticated regulatory network.

### 3.1. Transcriptional Regulation

**Figure 3:**
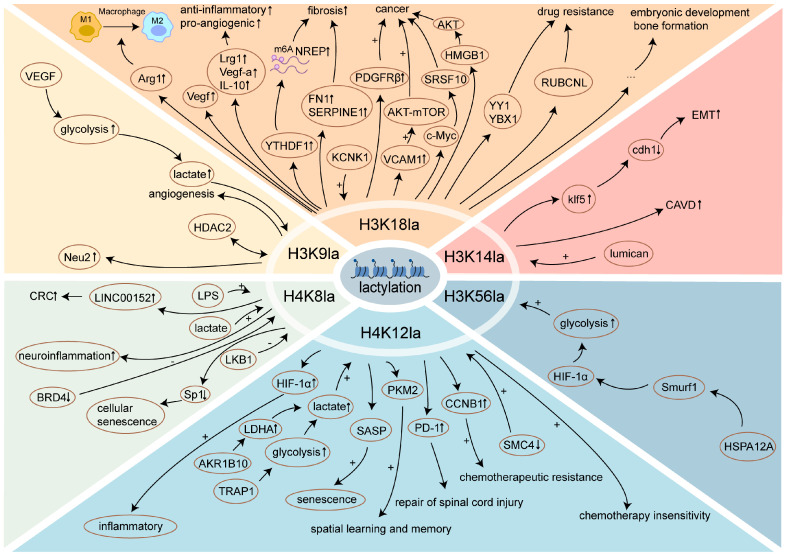
An overview of histone Kla sites and biological functions. Abbreviations: Arg1 (Arginase-1); Vegf (Vascular endothelial growth factor); Lrg1 (Leucine-rich alpha-2-glycoprotein 1); Il-10 (Interleukin-10); YTHDF1 (YTH domain-containing family protein 1); NREP (Neuronal protein 3.1); FN1 (Fibronectin 1); SERPINE1 (Serpin family E member 1); PDGFRβ (Platelet-derived growth factor receptor β); KCNK1 (Potassium two pore domain channel subfamily K member 1); VCAM1 (Vascular cell adhesion molecule 1); Akt (Protein Kinase B); mTOR (Mammalian target of rapamycin); HMGB1 (High mobility group box 1); SRSF10 (Serine/arginine-rich splicing factor 10); YBX1 (Y-box binding protein 1); RUBCNL (RUN domain and cysteine-rich domain containing, Beclin 1-interacting protein-like); Neu2 (Neuraminidase 2); CRC (Colorectal cancer); LPS (Lipopolysaccharide); LKB1 (Liver kinase B1); BRD4 (Bromodomain-containing protein 4); Sp1 (Specificity protein 1); HIF-1α (Hypoxia-inducible factor-1 alpha); PKM2 (Pyruvate kinase M2); CCNB1 (Cyclin B1); AKR1B10 (Aldo-keto reductase family 1 B10); PD-1 (Programmed cell death protein 1); SMC4 (Structural maintenance of chromosomes 4); TRAP1 (Tumour necrosis factor receptor-associated protein 1); SASP (Senescence-associated secretory phenotype); Smurf1 (SMAD specific E3 ubiquitin protein ligase 1); HSPA12A (Heat shock protein A12A); Klf5 (Krüppel-like factor-5); Cdh1 (Cadherin-1); EMT (Epithelial-mesenchymal transition); CAVD (Calcific aortic valve disease).

Histones, the earliest and most extensively studied lactylated proteins to date, serve as a representative model for investigating the functional mechanisms of Kla. Identified histone Kla sites include H3 at lysine9 (H3K9la), H3K14la, H3K18la, H3K27la, H3K56la, H4K8la, and H4K12la. Transcriptional regulation is a primary function of histone Kla, with Kla-mediated transcriptional programs modulating diverse physiological and pathological processes, such as macrophage phenotype regulation [18], tumor growth [19] and metastasis [20], progression of periodontitis [21], osteogenesis [22], osteoblast differentiation [23], development of preimplantation embryos [24], and meiosis in mouse oocytes [25] (Figure 3). The relationship between histone Kla and diseases will be elaborated in subsequent sections.

Histone Kla regulates gene transcription mainly by modifying chromatin structure, modulating transcription factor binding activity, and dynamically regulating the accessibility of gene promoter regions [26]. These transcriptional regulatory mechanisms are widely present across various cell types. H3K18la is recognized for its role in modulating macrophage polarization states through the direct up-regulation of reparative gene transcription, such as Arginase-1 (*Arg1*), Vascular endothelial growth factor (*Vegf*), Leucine-rich alpha-2-glycoprotein 1 (*Lrg1*), and Interleukin-10 (*Il-10*) [27,28,29]. The accumulation of lactate in Zeb1^+^ epithelioid cells leads to a significant upregulation in H3K18la, thereby increasing cellular chromatin accessibility and gene transcription [30]. H3K18la in alveolar epithelial cells during sepsis directly binds to the Methyltransferase-like 3 (*METTL3*) promoter region and promotes its transcription [31]. The hyper-H3K18la modification is also involved in the m6A methylation of neuronal protein 3.1 (Nrep) mRNA by enhancing the transcriptional activity of m6A readers, YTH domain-containing family protein 1(*YTHDF1*) [32]. In addition, *YTHDF2* [12] and Platelet-derived growth factor receptor β (*PDGFRβ*) [33] are both transcriptionally regulated by H3K18. The transcriptional control exerted by H3K18la renders it a promising target for clinical translation.

H4K12la is another important Kla site in histones. Studies have shown that the increased level of H4K12la promotes the transcription of Hypoxia-inducible factor-1 alpha (*HIF-1α*) [6], Senescence-associated secretory phenotype (*SASP*) [34], Cyclin B1 (*CCNB1*) [35], and Programmed cell death protein 1 (*PD-1*) [36]. In addition, the oncoprotein BRAF^V600E^ increases glycolytic flux to reprogram cellular Kla, leading to H4K12la-driven gene transcription and cell cycle dysregulation [37].

Transcriptional regulatory roles have also been identified for other histone Kla sites. H3K9la is significantly enriched in the promoter of LUC7-like protein 2 (*LUC7L2*), thereby activating the transcription and promoting the expression of *LUC7L2* [38]. H3K9la also leads to enhanced transcription of Neuraminidase 2 *(Neu2*), ultimately promoting muscle regeneration [39]. Lactate significantly increases the level of H4K8la at the LINC00152 promoter, leading to elevated expression of LINC00152 [40]. In addition, H3K14la enhances the transcription of Krüppel-like factor-5 (*klf5*), which binds to the Cadherin-1 (*cdh1*) promoter and inhibits its translation [41].

Beyond histones, Kla modification of non-histone proteins, particularly transcription factors (TFs), also plays a significant role in transcriptional regulation. The non-histone protein YY1, a widely studied TF, has been shown to undergo Kla at lysine 183 (K183), and hyperlactylated YY1 directly enhances Fibroblast growth factor 2 (*FGF2*) transcription [8]. Furthermore, YY1 Kla promotes the activation of microglia by regulating the transcription of a series of inflammatory genes, including *STAT3*, C-C chemokine receptor type 5 (*CCL5*), interferon regulatory factor 1 (*IRF1*), indoleamine 2,3-dioxygenase 1 (*IDO1*), and *SEMA4D* [9]. Another critical TF, IKAROS family zinc finger protein 1 (Ikzf1), undergoes Kla at Lys164, which promotes T_H_17 cell differentiation by directly upregulating key T_H_17-related genes such as runt-related transcription factor 1 (*Runx1*), toll-like receptor 4 (*Tlr4*), *IL2*, and *IL4* [42]. Methyl-CpG binding protein 2 (MECP2) has been reported to harbor multiple Kla sites, all of which exhibit transcriptional regulatory activity [43,44]. In addition, Centromere protein A (CENPA) Kla promotes the transcriptional activation of CENPA and synergizes with YY1 to drive the expression of cyclin D1 (*CCND1*) and neuropilin 2 (*NRP2*) [45]. With the continuous advancement of research, an increasing number of non-histone protein Kla sites with transcriptional regulatory functions have been identified. These modified proteins form intricate molecular networks with downstream regulated genes, participating in a wide range of cellular activities.

### 3.2. Metabolic Modulation

Kla is derived from the metabolite lactate, yet this modification reciprocally regulates metabolic activity. This metabolic circuitry is most frequently observed in glycolysis, particularly within tumor cells. Due to hypoxic conditions and high energy demands, tumor cells predominantly rely on glycolysis for energy production. The accumulated lactate drives elevated Kla levels, which promote the transcription of hypoxia-responsive proteins and directly or indirectly sustain glycolytic pathway activation. This metabolic reprogramming enhances tumor cell adaptation to hypoxia while ensuring a continuous energy supply. In pancreatic ductal adenocarcinoma (PDAC), the establishment of a glycolytic positive feedback loop is mechanistically dependent on the specific enrichment of H3K18la at the promoter regions of TTK protein kinase (TTK) and BUB1 mitotic checkpoint serine/threonine kinase B (BUB1B) genes, which subsequently activates their transcriptional activity and promotes PDAC progression [46]. In another study, researchers highlighted the pivotal role of Nucleolar and spindle-associated protein 1 (NUSAP1) Kla in regulating the glycolytic cycle in PDAC. Lactate upregulates NUSAP1 expression by inhibiting its proteasomal degradation through Kla modification, thereby establishing an NUSAP1-LDHA-glycolysis-lactate feedforward amplification loop [47]. However, in non-small cell lung cancer (NSCLC), histone Kla modification has been found to suppress glucose uptake and glycolysis in tumor cells [48], indicating the existence of more extensive and sophisticated regulatory mechanisms through which tumor cells utilize Kla-mediated modifications to orchestrate metabolic pathways. This metabolic regulation is also commonly observed in non-neoplastic diseases. A separate investigation revealed that hyperlactylation at histone H4K12 serves as an epigenetic driver of transcriptional reprogramming and subsequent glycolytic upregulation in Alzheimer’s disease (AD) pathogenesis [49]. Furthermore, another study reveals that H4K12la transcriptionally regulates the hypoxia-responsive protein HIF-1α, and this H4K12la-HIF-1α-glycolysis positive feedback loop plays a pivotal role in driving normal endometrial decidualization [50]. These findings suggest that Kla-mediated glycolytic reprogramming serves as a cellular defense mechanism, enabling cells to adapt to environmental challenges such as hypoxia and external stimuli, thereby revealing potential therapeutic targets for disease intervention.

### 3.3. Protein Structure and Activity

Kla modification plays a crucial regulatory role in maintaining protein structure and stability. As previously mentioned, NUSAP1 Kla is essential for preventing its degradation [47]. It has been reported that Kla modification can inhibit protein degradation by blocking ubiquitination [51]. Similar protein stabilization mechanisms have been reported in Kla modifications of LCP1 [52], β-catenin [53], and other proteins, suggesting that Kla may serve as a structural basis for protein stability and function.

Additionally, Kla modification can also regulate enzyme activity function. The glycolytic key enzyme pyruvate kinase M2 (PKM2) has been identified as a target of Kla, with the specific modification site at K62. PKM2 K62la suppresses its tetramer-to-dimer transition, thereby enhancing pyruvate kinase catalytic activity and diminishing nuclear translocation [54].

Kla can also modulate cellular signaling pathways to participate in the regulation of fundamental biological processes. However, it should be noted that this regulatory mechanism predominantly functions indirectly through Kla-mediated transcriptional control or protein interactions. For example, H4K12la enrichment at the NOD-like receptor protein 3 (NLRP3) promoter region activates its transcription, and the upregulated NOD-like receptor protein 3 (NLRP3) triggers downstream mammalian target of rapamycin (mTOR) pathway activation, ultimately leading to autophagic dysfunction in microglia [55]. Furthermore, lactate promotes the interaction between MOESIN and transforming growth factor-β (TGF-β) receptor I through Kla modification at lysine 72 (K72) of MOESIN protein, thereby potentiating downstream SMAD family member 3 (SMAD3) signaling activation [56]. Currently, research on Kla of signaling pathway proteins remains limited. Although lactylome analyses have identified numerous Kla modification sites on signaling molecules, their precise biological functions and regulatory mechanisms require further investigation.

### 3.4. Interactions Between Kla and Other PTMs

The crosstalk among PTMs has emerged as a prominent research focus, with growing evidence demonstrating potential synergistic or antagonistic effects between Kla and other PTMs. Among them, acetylation was the earliest PTM to be studied. Notably, Zhang et al. [1] initially discovered that histone Kla exhibits distinct temporal dynamics compared to acetylation. In mouse Kupffer cells, the subcellular distributions of proteins modified by lysine acetylation (Kac) and Kla exhibit distinct patterns, and the specific sequence motifs surrounding acetylated or lactylated lysine residues also demonstrate differential characteristics [57]. This opposite change may be related to competition between the two for the same modification site. Furthermore, the substrates involved in these two modifications, lactate and acetyl-CoA, exhibit potential competitive interactions within their metabolic synthesis pathways. However, a study finds that in macrophages of septic mice, lactate simultaneously upregulates both Kac and Kla levels of High mobility group box 1 (HMGB1), which is then secreted via exosomes, thereby increasing vascular endothelial permeability [58]. Additionally, similar to histone Kla, H3K27ac also plays a regulatory role in the immunosuppressive effects of macrophages [59]. These findings suggest the existence of more intricate interplays between Kac and Kla, and the precise mechanisms underlying their interactions await further in-depth investigation.

Kla may form cascade signaling networks with other PTMs to cooperatively regulate cellular activities. Under nutrient deprivation, UNC-51-like kinase 1 (ULK1) directly binds to LDHA and phosphorylates its serine-196 residue (Ser196), which promotes lactate production and subsequently modulates lactylated vacuolar protein sorting 34 (Vps34) levels [60]. A competitive regulatory relationship between Kla and ubiquitination has also been reported. The Kla modification at K91 of Transcription factor EB (TFEB) impairs its interaction with the E3 ubiquitin ligase WWP2, thereby suppressing TFEB ubiquitination and subsequent proteasomal degradation [51].

Kla and other acylations share similar enzymatic reaction mechanisms, suggesting a potential close relationship between them. A multi-omics study on nine PTMs (phosphorylation, acetylation, crotonylation, ubiquitination, lactylation, N-glycosylation, succinylation, malonylation, and β-hydroxybutyrylation) in hepatocellular carcinoma (HCC) further revealed the distribution patterns, modification preferences, and potential interactions between Kla and other acylations, providing a foundation for investigating PTM crosstalk [61]. However, under this macro perspective, the precise effects and regulatory mechanisms between Kla and other PTMs remain unclear, requiring further exploration and validation in future research.

## 4. The Role of Kla in Diseases

### 4.1. Kla in Cancer Biology

Kla plays a crucial role in cancer biology, primarily involved in tumorigenesis and metastasis, tumor immune microenvironment, and cancer therapy.

#### 4.1.1. Tumorigenesis and Metastasis

The pro-tumorigenic effect of H3K18la has been demonstrated in multiple cancer types, including neuroendocrine prostate cancer (PCa) [30], ocular melanoma [12], clear cell renal cell carcinoma [33], PDAC [46,47], breast cancer [62], and lung adenocarcinoma [63]. Transcriptional and metabolic regulation represent the common mechanism underlying H3K18la-mediated tumorigenesis. Specifically, H3K18la enhances chromatin accessibility, promoting cellular plasticity via transcriptional activation of neuroendocrine-associated genes and ultimately inducing neuroendocrine transdifferentiation in PCa [30]. Furthermore, the elevated levels of H3K14la and H3K18la in the promoter region of *SLC25A29* reduce the transcription of *SLC25A29*, thereby affecting the proliferation, migration, and apoptosis of endothelial cells [63]. H3K18la also drives the transcriptional activity of vascular cell adhesion molecule 1 (*VCAM1*) and then activates the protein kinase B (AKT)- mTORsignaling pathway, thereby promoting gastric cancer development [64]. H3K18la modification can also promote the transcriptional expression of non-coding RNAs. The activated NF-κB pathway can promote lactate production through the Warburg effect, leading to increased levels of H3K18la at the promoter region of LINC01127, which is conducive to its expression. LINC01127 regulates the self-renewal of cancer cells through the MAP4K4/JNK/NF-κB axis [65]. Similar transcriptional regulatory mechanisms have also been reported for other histone Kla sites, thereby contributing to tumor progression [66].

**Figure 4:**
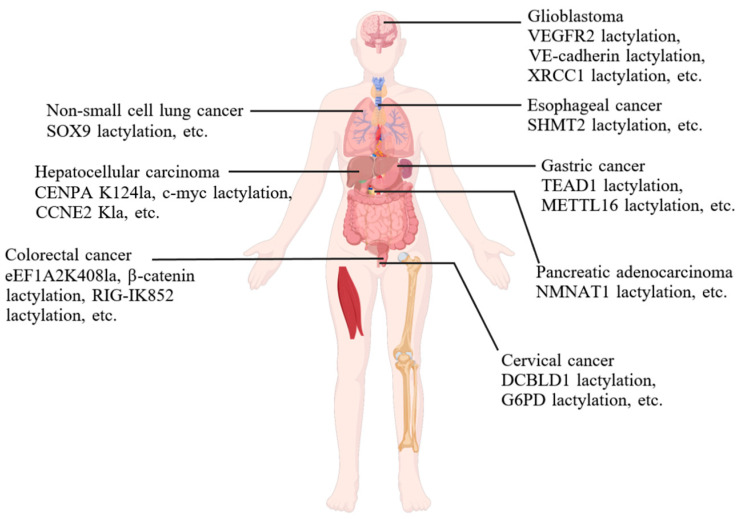
The relationship between non-histone Kla and cancers. Abbreviations: SOX9 (SRY-box transcription factor 9); CENPA (Centromere protein A); CCNE2 (Cycline2); eEF1A2 (Elongation factor 1 alpha 2); RIG-I (Retinoic acid-inducible gene 1); VEGFR2 (Vascular endothelial growth factor receptor 2); VE-cadherin (Vascular endothelial cadherin); XRCC1 (X-ray repair cross-complementing protein 1); SHMT2 (Serine hydroxymethyl transferase 2); TEAD1 (TEA domain transcription factor 1); METTL16 (Methyltransferase-like 16); NMNAT1 (Nicotinamide mononucleotide adenylyltransferase 1); DCBLD1 (Discoidin, CUB, and LCCL domain-containing type I); G6PD (Glucose-6-phosphate dehydrogenase).

Existing studies have demonstrated that non-histone Kla plays a crucial regulatory role in various cancers (Figure 4). In contrast to histones, the diversity of non-histone proteins renders the regulatory mechanisms of non-histone Kla in tumor progression more complex. Gene expression regulation represents one of the key mechanisms. Centromere proteins (CENPs) are an important mitosis-related protein complex involved in kinetochore assembly and chromosome segregation. CENPA K124la enhances its transcriptional activation in HCC cells, acting as a transcriptional regulatory factor to promote the occurrence of HCC by cooperating with YY1 [45]. KAT8-mediated Kla is also a molecular mechanism that promotes tumor growth. The KAT8-mediated eEF1A2 K408la is a functional hotspot for regulating translation elongation, which contributes to the occurrence of tumors [11]. In gastric cancer cells, alanyl-tRNA synthetase 1 (AARS1) translocates into the nucleus, directly catalyzing the Kla of Yes-associated protein (YAP) at position K90 and TEA domain transcription factor 1 (TEAD1) at position K108, thereby activating the expression of downstream target genes and promoting the proliferation of tumor cells [67]. Serine hydroxymethyl transferase 2 (SHMT2) is involved in esophageal cancer progression by interacting with Methylenetetrahy-drofolate Dehydrogenase 1 Like (MTHFD1L), while hypoxia-induced SHMT2 Kla in turn enhanced MTHFD1L expression and accelerated the malignant progression of EC cells [68].

Furthermore, non-histone Kla contributes to tumorigenesis by modulating protein stability and function, particularly of some key metabolic enzymes. Hyperactive Wnt/β-catenin signaling is implicated in the initiation and progression of various types of cancer [69]. Hypoxia treatment dramatically increased the Kla level of β-catenin in colorectal cancer (CRC) cells, which further enhanced the protein stability and expression of β-catenin, thus aggravating the proliferation and stemness of cancer cells [53]. AARS1 lactylates p53 at specific lysine residues (K120 and K139), which impairs p53’s DNA binding capacity and liquid–liquid phase separation (LLPS), thereby inhibiting the tumor-suppressing function of p53 [70]. Lactate enhances the Kla level of Nicotinamide mononucleotide adenylyltransferase 1 (NMNAT1), promoting its nuclear translocation and maintaining enzymatic activity. Together, these processes support the intracellular NAD^+^ salvage pathway within the cell nucleus, preventing NAD^+^ depletion, and activating silent information regulator sirtuin 1 (SIRT1). As a result, cellular stress is reduced, which helps sustain the survival of PDAC cells under glucose-deprived conditions [71]. Furthermore, lactate inhibits Discoidin, CUB, and LCCL domain-containing type I (DCBLD1) degradation by directly increasing DCBLD1 Kla. DCBLD1 inhibited glucose-6-phosphate dehydrogenase (G6PD) autophagic degradation, activating the pentose phosphate pathway to promote cervical cancer progression [72]. Furthermore, high-risk human papillomavirus 16 E6 (HPV16 E6) inhibits G6PD K45la to increase the enzymatic activity of G6PD. The activation of G6PD mediated by human papillomavirus 16 E6 (HPV16 E6) is crucial for the growth of cancer cells both in vitro and in vivo [73]. With the widespread application of lactylome analysis, more and more protein Kla are identified, which provides a theoretical foundation for identifying potential therapeutic targets against tumors in future research.

#### 4.1.2. Tumor Immune Microenvironment

Tumor microenvironment refers to the close relationship between the occurrence, growth, and metastasis of tumors and the internal and external environments in which the tumor cells reside, especially the interaction between the cancer cells and the immune system. It has been demonstrated that Kla modification can regulate tumor progression by affecting the function of immune cells. Among these, tumor-associated macrophages (TAMs) have been the most extensively studied. The currently widely accepted theory posits that lactate produced by tumor cells via the Warburg effect is taken up by macrophages. Lactate-mediated histone and non-histone Kla modifications then drive the polarization of macrophages toward a pro-tumor phenotype, thereby contributing to the regulation of the tumor immune microenvironment [74]. The lactate produced by the tumor stimulates H3K18la, which inhibits the transcription of the retinoic acid receptor γ (RARγ) gene in macrophages. This leads to an increase in the levels of IL-6 in the tumor microenvironment, and by activating the signal transducer and activator of the transcription 3 (STAT3) signaling pathway, it confers upon macrophages the ability to promote tumor growth [75]. Accumulated lactate in the tumor microenvironment effectively induces the upregulation of METTL3 in tumor-infiltrating myeloid cells (TIMs) through H3K18la, and Kla modification on the zinc-finger region of *METTL3* boosts its ability to bind m6A-modified RNA. The regulatory mechanism involving Kla-METTL3- Janus kinase (JAK1) -STAT3 strongly promotes the immunosuppressive capabilities of TIMs [76].

Kla-mediated regulation in other immune cell types has also been documented. orphan G protein-coupled receptor 37 (GPR37) boosts the expression of LDHA and glycolysis by triggering the hippo signaling pathway, which results in an elevation of H3K18la and the upregulation of cytokines that are involved in the recruitment of neutrophils, such as chemokines 1 (CXCL1) and CXCL5, thereby facilitating the progression of cancer [77]. The H3K18la enrichment in the promoter region of *ATXN7* activates the transcription of circATXN7. CircATXN7 sequesters the NF-κB p65 subunit in the cytoplasm, making tumor-specific cytotoxic T lymphocytes sensitive to activation-induced cell death [78]. Lactate also regulates Treg cells through Kla of MOESIN at the Lys72 residue, thereby leading to the occurrence of tumors [56]. Moreover, *Escherichia coli* induces Kla of the retinoic acid-inducible gene 1 (RIG-I), which reduces the activation of MAVS and the downstream NF-κB pathway. This leads to a decrease in NLRP3 activation, promoting macrophage M2 polarization and facilitating the differentiation of T regulatory cells. These effects contribute to immune suppression, thereby promoting tumor progression [79]. It is noteworthy that the spatial distribution and functional implications of Kla in diverse tumor-associated immune cells have not been fully elucidated. Specifically, whether lactate-induced Kla serves as an intercellular communication mediator between tumor cells and immune cells, or among immune cell subsets, warrants further exploration.

#### 4.1.3. Cancer Therapy

Kla plays a pivotal regulatory role in cancer therapy, particularly by mediating mechanisms of resistance to chemotherapy and immunotherapy. A study reveals that H3K18la promotes cisplatin resistance in bladder cancer by upregulating the expression of YY1 or Y-box binding protein 1, while the specific regulatory mechanisms have not been thoroughly investigated [80]. Tumor-derived lactate promotes the expression of the autophagy-enhancing protein RUN domain and cysteine-rich domain containing, VEGF-interacting protein-like (RUBCNL) through H3K18la in CRC, thereby promoting resistance to bevacizumab treatment [81]. H3K18la increases MYC activity and PD-1 expression by directly activating the transcription of POM121, leading to poor prognosis in patients [82]. The above data suggest that H3K18la is one of the key mechanisms of drug resistance in tumor cells, which also provides new strategies for cancer treatment.

Aldo-keto reductase family 1 B10 (AKR1B10), as a precursor that stimulates H4K12la, activates the transcription of CCNB1 and accelerates DNA replication and cell cycle progression. The AKR1B10/glycolysis/H4K12la/CCNB1 pathway promotes chemoresistance to pemetrexed chemotherapy [35]. Furthermore, H4K12la results in insensitivity to the chemotherapy drug irinotecan, and this insensitivity is associated with an increase in Kla levels [83]. H3K9la activates the transcription and promotes the expression of LUC7L2. LUC7L2 mediates the retention of intron 7 of MLH1, thereby reducing the expression of MLH1, which in turn inhibits mismatch repair, ultimately leading to temozolomide resistance in glioblastoma multiforme [38].

Cancer cells can rapidly repair damaged DNA with lactate after chemotherapy-induced DNA damage, thereby reducing the therapeutic effect and leading to drug resistance. NBS1 K388la facilitates the formation of the Meiotic recombination 11 homolog (MRE11)-RAD50-NBS1 complex and the accumulation of homologous recombination (HR) repair proteins at DNA double-strand break sites, resulting in resistance to chemotherapy [10]. Additionally, MRE11 is lactylated following DNA damage. MRE11 Kla exerts a key function in regulating MRE11 DNA-binding ability and subsequent DNA end resection. High MRE11 Kla level promotes homologous recombination and chemoresistance in cancer cells [84]. The accumulation of lactate increases K247la on X-ray repair cross-complementing protein 1 (XRCC1), which enhances DNA repair through its increased nuclear localization, contributing to the therapeutic resistance of glioblastoma stem cells [85]. Additionally, certain clinically approved drugs or natural compounds have been reported to modulate disease progression through Kla-related mechanisms, which will be elaborated on in later sections. In summary, current research demonstrates the significant clinical translational value of Kla studies. Biomarkers and therapeutic strategies developed based on Kla modification show promising potential as novel approaches for disease diagnosis and treatment.

### 4.2. Kla in Inflammation and Infection

Macrophages serve as central regulators of inflammatory responses and host defense against infections. The role of H3K18la-mediated macrophage polarization in inflammation and infection has been well established, demonstrating regulatory mechanisms analogous to those observed in tumors [27]. In addition to histones, non-histone protein Kla also plays regulatory roles in macrophage functional modulation. PKM2 Kla inhibits its tetramer-to-dimer transition, promotes its pyruvate kinase activity, and reduces nuclear distribution, mediating the transition of macrophage phenotype [54]. In macrophages, the lactylated HMGB1 is transferred from the nucleus to the cytoplasm, and then secreted and released into the circulation through exosomes. The secreted exosomal HMGB1 further disrupts endothelial integrity and increases vascular permeability. Reducing circulating exosomal HMGB1 benefits patients with polymicrobial sepsis [58].

Furthermore, hyperlactylation of Ikzf1 at Lys164 promoted TH17 differentiation by directly modulating the expression of T_H_17-related genes, thereby aggravating experimental autoimmuneuveitis inflammation [42]. Additionally, a study has shown that Sox10 Kla is activated in a phosphorylation-dependent manner, participating in the transcriptional program of vascular smooth muscle cell (VSMC) transdifferentiation, promoting pyroptosis and maintaining vascular inflammation [86]. YY1 Kla also promotes the activation of microglia by regulating the transcription of a series of inflammatory genes, including *STAT3*, *CCL5*, *IRF1*, *IDO1*, and *SEMA4D* [9]. In addition, Kaposi’s sarcoma-associated herpesvirus (KSHV) polyadenylated nuclear RNA (PAN) orchestrated N-acetyltransferase 10 (NAT10) and α-tubulin acetyltransferase 1 (ATAT1) to enhance NAT10 Kla, resulting in tRNA^Ser-CGA−1−1^ N^4^-acetylcytidine modification, eventually boosting KSHV reactivation [87]. Current research on Kla in inflammation and infection remains predominantly focused on immune cells, while the Kla profiles of other cell types in these pathological contexts warrant further investigation.

### 4.3. Kla in Cardiovascular Diseases

H3K18la has been demonstrated to be associated with arterial calcification [88] and atherosclerosis [89]. H3K56la is initially found during myocardial ischemia/reperfusion injury and plays a role in improving cardiomyocyte survival [90]. In addition, lumican facilitates the development of aortic valve calcification via H3K9la and H3K14la [91]. These studies highlight the critical role of histone Kla in cardiovascular diseases.

In cardiomyocytes, increased Kla level promotes YTHDF2 expression where YTHDF2 mechanistically contributes to cardiomyocytes’ cell size change and apoptosis in an RNA-m6A-independent manner [92]. Furthermore, the exercise-induced atheroprotective effect requires an interaction between Mecp2k271la and H3K36me3, leading to increased chromatin accessibility and transcriptional repression of RUNX1 [93]. The α-myosin heavy chain (α-MHC) K1897la regulates the interaction between α-MHC and Titin, and the decrease in α-MHC K1897la predisposes to heart failure [94].

### 4.4. Kla in Neurological Disorders

There are literature reports on the presence and role of Kla in neurological disorders [95]. In naturally aging mice and AD model mice, both H3K18la and pan-Kla are significantly upregulated in senescent microglia and hippocampal tissues. Enhanced H3K18la directly stimulates the NFκB signaling pathway by increasing its binding to the Rela (p65) and NFκB1 (p50), thereby upregulating the components of the SASP, IL-6, and IL-8 [96]. The reduction in H4K8la induced by Bromodomain-containing protein 4 (BRD4) silencing exacerbates the A1 polarization of astrocytes and increases neuroinflammation and neuronal death, ultimately impairing the recovery and prognosis of neurological function in mice after subarachnoid hemorrhage [97]. H4K12la also plays a role in neural development. A positive feedback mechanism involving glycolysis, H4K12la, and PKM2 in microglia promotes the progression of AD [49]. Additionally, the increase in H4K12la promotes the transcription of PD-1, thereby facilitating the repair of spinal cord injury [36].

Furthermore, the elevated Kla level of ADP-ribosylation factor 1 exacerbates cerebral I/R injury [98]. The Kla of synaptosome-associated protein 91 (SNAP91) enhances the formation of synaptic structures and neuronal activity in the medial prefrontal cortex, conferring resilience to chronic restraint stress, and can prevent anxiety-like behaviors in chronic restraint stress mice [99]. The enhancement of METTL3 Kla level further increases the stability and the expression levels of METTL3, which further inhibits ferroptosis by regulating the levels of transferrin receptors during the process of cerebral hemorrhage [100].

### 4.5. Kla in Other Diseases

H3K18la also facilitates the development of fibrosis. The hyper-H3K18la modification is involved in the m6A methylation of Nrep mRNA by enhancing the transcriptional activity of m6A readers, YTHDF1, thereby contributing to the progression of idiopathic pulmonary fibrosis [32]. In hepatic stellate cells, lactate derived from hexokinase 2 (HK2) promotes histone Kla, while HK2 deficiency inhibits H3K18la and alleviates hepatic stellate cell activation [101]. High expression of fibrosis-related genes regulated by H3K18la is observed in hypoxia-induced fibrosis [102]. Furthermore, the increase in scleral glycolytic lactate levels promotes the expression of Notch1 through H3K18la, thereby inducing fibroblast-to-myofibroblast trans-differentiation [103].

The Kla of fatty acid synthase (FASN) K673 significantly inhibits the activity of FASN and reduces the accumulation of lipid in hepatocytes, which plays an important role in the regulation of hepatic lipid metabolism by mitochondrial pyruvate carrier [104]. FASN Kla also reduces the activity of high-intensity interval training, thereby inhibiting the generation of new fat [105]. Additionally, in liver injury, treatment with acetaminophen inhibits the SIRT1/Peroxisome Proliferator-Activated Receptor Gamma Coactivator 1-Alpha (PGC-1α)/LDHB axis, and increases mitochondrial lactate levels and protein Kla levels, ultimately promoting pathological damage in the liver. Activation of the PGC-1α/LDHB axis alleviates liver injury by reducing lactate production [106]. Mitochondrial dysfunction and enhanced anaerobic glycolysis of glucose lead to lactate accumulation, which is involved in the progression of diabetic nephropathy [107]. The mitochondrial localization of acyl-CoA synthetase family member 2 (ACSF2) K182la can promote mitochondrial dysfunction in high-glucose-treated HK-2 cells, potentially leading to diabetic nephropathy [108]. Glutamine could prevent intervertebral disc degeneration by glycolysis inhibition-decreased adenosine-5’-monophosphate-activated protein kinase α (AMPKα) Kla, which promotes autophagy and suppresses nucleus pulposus cell senescence [109]. Current evidence demonstrates that Kla is a ubiquitous modification across diverse pathologies, while its pathophysiological roles and mechanistic underpinnings in broader disease spectra remain to be systematically investigated.

## 5. Clinical Translation of Kla

Kla establishes a close connection between cellular metabolism and gene expression regulation, and plays a crucial role in the occurrence and development of various diseases. Research on Kla can provide new ideas and approaches for clinical practice.

### 5.1. Biomarker Development

Lactate is associated with a wide range of diseases, including cardiovascular diseases, respiratory system diseases, cancer, inflammation, and fibrosis. Previous clinical studies have shown that ventricular lactate levels are positively correlated with neurological impairment [110]. In addition, lactate has been recognized as a biomarker for respiratory chain diseases [111]. This proves that the level of lactate is related to the poor prognosis of diseases and can serve as a biomarker for various diseases. Previous clinical studies have shown that lactate itself is a marker of poor prognosis in sepsis [112]. Further research has found that the Kla level induced by lactate is positively correlated with the poor prognosis of sepsis, suggesting that Kla is also a potential biomarker for sepsis [113]. The above research indicates that lactate and Kla can suggest the progression of various diseases as well as poor prognoses, and can serve as potential biomarkers for monitoring the disease course in clinical practice.

### 5.2. Kla-Targeted Therapeutic Interventions

Studies have demonstrated that lactate-derived Kla plays a pivotal role in disease progression. Therefore, targeted modulation of the glycolytic pathway may represent a crucial translational avenue for clinical applications of Kla. LDH is one of the key enzymes in the glycolytic pathway, which can catalyze the reduction of pyruvate to produce lactate. There is experimental evidence proving that oxamate, an LDHA inhibitor, can reduce chondrocyte apoptosis [114]. Oxamate suppressed the generation of lactate and reduced the activity levels of the gene promoters for CD39, CD73, and chemokine (C-Cmotif) receptor 8 (CCR8) by decreasing the H3K18la, thereby improving the efficacy of chimeric antigen receptor (CAR)-T cell therapy in glioblastoma [115]. In addition, the study found that Genistein inhibits glycolysis and induces cell apoptosis by suppressing the expression and activity of HIF-1α [116]. The role of dexamethasone in controlling asthma is related to its inhibition of the HIF-1α glycolysis-lactate axis and the subsequent protein Kla, particularly in macrophages [117]. These drugs affect diseases by influencing the glycolytic pathway, which holds clinical research significance.

Notably, suppression of lactate uptake similarly regulates Kla levels. Experimental evidence demonstrates that pharmacological inhibition of MCT4 upregulates Kla at the K1897 site of the α-myosin heavy chain (α-MHC), consequently ameliorating cardiac dysfunction [94], highlighting the therapeutic potential of lactate uptake inhibitors in clinical translation.

Furthermore, researchers have designed a cell-penetrating peptide (CPP) to target the Kla of MRE11 K673. This peptide presents evident inhibition of MRE11 K673la and impairs HR, which in turn promotes cancer cell sensitivity to chemotherapy [84]. This indicates that by blocking Kla sites through CPP, it is possible to regulate tumor progression and the efficacy of chemotherapy. This may be one of the directions for future clinical applications.

Some drugs and natural compounds can also treat diseases by influencing the Kla mechanism (Table 1). Enzalutamide has demonstrated significant efficacy in the treatment of advanced PCa. Long-term Enzalutamide treatment leads to the up-regulation of SLC4A4, which in turn mediates P53 Kla via the NF-κB/STAT3/SLC4A4 axis, ultimately leading to the development of Enzalutamide resistance and progression of PCa [118]. Gambogic acid facilitates the recruitment of the dehydrolase SIRT1, which leads to the removal of Kla on Canopy FGF signaling regulator (CNPY3). This process ultimately results in the functional and structural disruption of lysosomal proteins, thereby inducing pyroptosis [119]. Besides, 20 (S)-ginsenoside Rh2 (GRh2) ameliorated drug resistance by downregulating the Kla level and directly inhibiting METTL3 [120]. Evolodiamine significantly blocks lactate-induced angiogenesis by restricting histone Kla and the expression of HIF-1α, further enhancing the transcription of Sema3A and inhibiting the transcription of PD-L1 [121]. In addition, the antimalarial drug artemisinin targets p300-mediated PKM2 Kla allosterically, exerting a significant anti-proliferative effect on pathological fibroblast-like synoviocytes and is expected to be a potential therapeutic intervention for rheumatoid arthritis [122]. In summary, the Kla mechanism provides a theoretical foundation for the clinical application of these drugs or natural compounds, and further clinical studies are warranted to validate their therapeutic potential.

## 6. Challenges in Kla Research

The rapid advancement of Kla research demonstrates its tremendous clinical potential, yet undeniable challenges persist that hinder both mechanistic exploration and clinical translation.

The initial discovery of Kla modification was achieved through high-performance liquid chromatography–tandem mass spectrometry (HPLC-MS/MS) technology [1]. A subsequent study reported a tandem mass spectrometry detection technique based on the cyclic immonium ion of lactyllysine, which significantly enhanced the sensitivity and specificity of Kla detection [123]. In current research, the term “lactylation” typically refers to L-lactylation (K_l-la_) derived from L-lactate. However, this modification actually exists in three isomeric forms: K_l-la_, N-ε-(carboxyethyl)-lysine (K_ce_), and D-lactyl-lysine (K_d-la_). To address this complexity, researchers have developed two novel approaches: (1) chemical derivatization coupled with HPLC for separation of these isomers, and isomer-specific antibodies for identification [124]. These technological advancements not only substantially expand the lactylproteome landscape but also provide a robust approach to discriminate Kla from structurally similar acyl modifications. In addition, Sun et al. [125] developed an alkynyl-functionalized bioorthogonal chemical reporter, YnLac, which enables the detection and identification of protein Kla, providing a powerful chemical tool for Kla research. It is worth noting that computational models developed using automated machine learning have been demonstrated to be a powerful analytical tool for predicting Kla modifications [126]. Although significant progress has been made in Kla detection and analysis technologies, several challenges remain. Firstly, the detection of Kla at single-cell and live-cell levels has not yet been fully achieved, which hinders in-depth mechanistic studies. Secondly, current Kla detection methods remain confined to laboratory research. Developing rapid, cost-effective, and accurate detection techniques for clinical applications will require extensive further exploration.

Several critical knowledge gaps remain in the mechanistic studies of Kla. Primarily, the enzymatic system regulating Kla has not been fully characterized, hindering a comprehensive understanding of its molecular mechanisms. Secondly, Kla interacts with other PTMs to form an intricate cellular regulatory network, yet the precise operational principles governing these interactions remain elusive. Future investigations should elucidate competitive inhibition and synergistic effects among different PTMs, and decipher how these modifications precisely regulate gene expression and metabolic reprogramming across diverse cell types and physiological states. Finally, the functional characterization of Kla remains incomplete. Beyond histone Kla, the biological functions and regulatory mechanisms of non-histone protein Kla demand a more systematic investigation.

Currently, site-directed mutagenesis remains the gold standard for validating the functional roles of Kla sites, primarily through lysine-to-arginine (K-to-R) mutations or Kla-mimicking glutamine substitutions (K-to-Q). However, this field faces several critical challenges: First, current approaches fail to fully recapitulate the dynamic and reversible nature of Kla modifications, particularly their rapid regulation in response to cellular metabolic fluctuations. Furthermore, for proteins with multiple cooperative Kla sites, single-site mutation strategies often cannot capture the true biological complexity. Future directions should focus on developing more precise genetic tools, establishing protein engineering methods that better mimic the dynamic features of Kla, and integrating single-cell sequencing with live-cell imaging technologies to resolve the spatiotemporal effects of these mutations.

## 7. Conclusions

Kla is an extensively widespread form of protein modification. This review synthesizes newly discovered Kla sites on histones and non-histones, and the latest advances in mechanisms. Drugs that regulate protein Kla levels may have the potential to block or delay disease progression and tumor resistance. However, despite the identified functions of Kla in various biological processes, its application in clinical practice still faces huge challenges. Future studies are still required to elucidate the precise functions and underlying mechanisms of Kla, which will facilitate the development of novel diagnostic and therapeutic strategies for clinical applications.

## Figures and Tables

**Table 1:** Targeted therapeutic drugs for protein Kla and their efficacy.

Medicine	Target	Function	Disease	Reference
Oxamate	LDHA	Apoptosis ↓	Osteoarthritis	[114]
	LDHA	Immunosuppression of TME ↓	Glioblastoma multiforme	[115]
Genistein	HIF-1α	Tumour cell growth ↓	HCC	[116]
Dexamethasone	HIF-1α	Pyroptosis ↑	Asthma	[117]
CPP	MRE11 K673la	cisplatin and PARPi sensitivity ↑	Breast cancer	[84]
Gambogic acid	CNPY3	Pyroptosis ↑	PCa	[119]
GRh2	METTL3	all-trans retinoic acid resistance ↓	promyelocytic leukemia	[120]
Evodiamine	Kla, HIF-1α	Angiogenesis ↓	PCa	[121]
Artemisinin	PKM2	cell proliferation ↓	rheumatoid arthritis	[122]

The downward arrow (↓) indicates inhibition of the function, while the upward arrow (↑) denotes activation/promotion of the function. Abbreviations: CPP (Cell-penetrating peptide); GRh2 (20 (S)-ginsenoside Rh2); MRE11 (Meiotic recombination 11 homolog); CNPY3 (Canopy FGF signaling regulator); TME (Tumor microenvironment); PARPi (Poly [ADP-ribose] polymerase inhibitors); HCC (Hepatocellular carcinoma); PCa (Prostate cancer).

## Data Availability

No data were used for the research described in the article.

## Abbreviations

The following abbreviations are used in this manuscript:

Kla	Lysine lactylation
MCT	Monocarboxylate transporters
BMDMs	Bone marrow-derived macrophages
YTHDF1	YTH domain-containing family protein 1
HK2	hexokinase 2
VHL	Von Hippel–Lindau
PDGFRβ	Platelet-derived growth factor receptor β
m1A	N1-methyladenosine
PML	Promyelocytic leukemia protein
VCAM1	Vascular cell adhesion molecule 1
BUB1B	BUB1 mitotic checkpoint serine/threonine kinase B
LDH	Lactate dehydrogenase
PDAC	Pancreatic ductal adenocarcinoma
KCNK1	Potassium two pore domain channel subfamily K member 1
SRSF10	Serine/arginine-rich splicing factor 10
MDM4	Murine double minute 4
Bcl-x	BCL2-like 1
HMGB1	High-mobility group box 1
BZW2	Basic leucine zipper and W2 domains 2
PD-1	Programmed cell death protein 1
YY1	Yin Yang-1
STAT3	Signal transducer and activator of transcription 3
TIMs	Tumor-infiltrating myeloid cells
SASP	Senescence-associated secretory phenotype
CAR	Chimeric antigen receptor
VSMCs	Vascular smooth muscle cells
AD	Alzheimer’s disease
HDAC2	Histone deacetylase 2
CENPs	Centromere proteins
AARS1	Alanyl-tRNA synthetase 1
LLPS	Liquid–liquid phase separation
CCNE2	Cycline2
NSCLC	Non-small cell lung cancer
RIG-I	Retinoic acid-inducible gene 1
SHMT2	Serine hydroxymethyl transferase 2
G6PD	Glucose-6-phosphate dehydrogenase
HPV16 E6	High-risk human papillomaviruse 16 E6
VE-cadherin	Vascular endothelial cadherin
HR	Homologous recombination
CPP	Cell-penetrating peptide
VEGF	Vascular endothelial growth factor
EphA2	Erythropoietin-producing hepatocellular A2
ARSI	Androgen receptor signaling inhibitor
MAPK	Mitogen-activated protein kinase
FASN	Fatty acid synthase
PAN	Polyadenylated nuclear RNA
NAT10	N-acetyltransferase 10
ATAT1	α-tubulin acetyltransferase 1
CCNB1	Cyclin B1
AKR1B10	Aldo-keto reductase family 1 B10
LUC7L2	LUC7-like protein 2
PCa	Prostate cancer
MRE11	Meiotic recombination 11 homolog
XRCC1	X-ray repair cross-complementing protein 1
Ikzf1	IKAROS family zinc finger protein 1
HPLC-MS/MS	High-performance liquid chromatography-tandem mass spectrometry
α-MHC	α-myosin heavy chain
PTMs	Post-translational modifications
TFs	Transcription factors
CENPA	Cyclin D1
NRP2	Neuropilin 2
TGF-β	Transforming growth factor-β
Kac	Lysine acetylation
ULK1	UNC-51-like kinase 1
TFEB	Transcription factor EB
CRC	Colorectal cancer
TAMs	Tumor-associated macrophages
KSHV	Kaposi’s sarcoma-associated herpesvirus
PAN	Polyadenylated nuclear RNA
NAT10	N-acetyltransferase 10
ATAT1	α-tubulin acetyltransferase 1
GRh2	20(S)-ginsenoside Rh2
K_l-la_	L-lactylation
K_ce_	N-ε-(carboxyethyl)-lysine
K_d-la_	D-lactyl-lysine
HPV16 E6	Human papillomavirus-16 E6
ACSS2	Acetyl-CoA synthetase 2
TIP60	Tat-interactive protein 60 kDa
Brg1	Brahma-related gene 1
SIRT3	Sirtuin 3
HDAC	Histone deacetylase
Lrg1	Leucine-rich alpha-2-glycoprotein 1
Il-10	Interleukin-10
NREP	Neuronal protein 3.1
Akt	Protein Kinase B
mTOR	Mammalian target of rapamycin
RUBCNL	RUN domain and cysteine-rich domain containing, Beclin 1-interacting protein-like
Neu2	Neuraminidase 2
SMC4	Structural maintenance of chromosomes 4
BRD4	Bromodomain-containing protein 4
eEF1A2	Elongation factor 1 alpha 2
TEAD1	TEA domain transcription factor 1
DCBLD1	Discoidin, CUB, and LCCL domain-containing type I
CNPY3	Canopy FGF signaling regulator
ATP	Adenosine triphosphate
SNAP91	Synaptosome-associated protein 91
p300	Histone acetyltransferase
KAT8	Lysine acetyltransferase 8
Cdh1	Cadherin-1
FGF2	Fibroblast growth factor 2
CCL5	C-C chemokine receptor type 5
IRF1	Interferon regulatory factor 1
IDO1	Indoleamine 2,3-dioxygenase 1
Tlr4	Toll-like receptor 4
Runx1	Runt-related transcription factor
TTK	TTK protein kinase
BUB1B	BUB1 mitotic checkpoint serine/threonine kinase B
Vps34	Vacuolar protein sorting 34
NBS1	Nijmegen Breakage Syndrome protein 1
YAP	Yes-associated protein
MTHFD1L	Methylenetetrahy-drofolate Dehydrogenase 1 Like
NMNAT1	Nicotinamide mononucleotide adenylyltransferase 1
SMAD3	SMAD family member 3
RARγ	Retinoic acid receptor γ
JAK1	Janus kinase 1
GPR37	Orphan G protein-coupled receptor 37
CXCL1	Chemokines 1
NLRP3	NOD-like receptor protein 3
PGC-1α	Peroxisome proliferator-activated receptor gamma coactivator 1-Alpha
SIRT1	Silent information regulator sirtuin 1
ACSF2	Acyl-CoA synthetase family member 2
AMPKα	Adenosine-5’-monophosphate-activated protein kinase α
CCR8	Chemokine (C-Cmotif) receptor 8
